# WtsWrng Interim Comparative Effectiveness Evaluation and Description of the Challenges to Develop, Assess, and Introduce This Novel Digital Application in a Traditional Health System

**DOI:** 10.3390/ijerph192113873

**Published:** 2022-10-25

**Authors:** Laura Sampietro-Colom, Carla Fernandez-Barcelo, Ismail Abbas, Blanca Valdasquin, Nicolau Rabasseda, Borja García-Lorenzo, Miquel Sanchez, Mireia Sans, Noemi Garcia, Alicia Granados

**Affiliations:** 1 Assessment of Innovations and New Technologies Unit, Research and Innovation Directorate, Clínic Barcelona University Hospital, 08036 Barcelona, Spain; 2 Mangrana Ventures S.L., 08006 Barcelona, Spain; 3 Kronikgune Institute for Health Sciences Research, 48902 Barakaldo, Spain; 4 Emergency Department, Clínic Barcelona University Hospital, 08036 Barcelona, Spain; 5 CAP Comte Borrell, Consorci Atenció Primaria Salut Barcelona Esquerra—CAPSBE, 08029 Barcelona, Spain; 6 Health 2.0 Section of the Col·Legi Oficial de Metges de Barcelona, 08017 Barcelona, Spain

**Keywords:** mHealth, innovation, technology assessment, digital health, digital application, triage, WtsWrng

## Abstract

Science and technology have evolved quickly during the two decades of the 21st century, but healthcare systems are grounded in last century’s structure and processes. Changes in the way health care is provided are demanded; digital transformation is a key driver making healthcare systems more accessible, agile, efficient, and citizen-centered. Nevertheless, the way healthcare systems function challenges the development (Innovation + Development and regulatory requirements), assessment (methodological guidance weaknesses), and adoption of digital applications (DAs). WtsWrng (WW), an innovative DA which uses images to interact with citizens for symptom triage and monitoring, is used as an example to show the challenges faced in its development and clinical validation and how these are being overcome. To prove WW’s value from inception, novel approaches for evidence generation that allows for an agile and patient-centered development have been applied. Early scientific advice from NICE (UK) was sought for study design, an iterative development and interim analysis was performed, and different statistical parameters (Kappa, B statistic) were explored to face development and assessment challenges. WW triage accuracy at cutoff time ranged from 0.62 to 0.94 for the most frequent symptoms attending the Emergency Department (ED), with the observed concordance for the 12 most frequent diagnostics at hospital discharge fluctuating between 0.4 to 0.97; 8 of the diagnostics had a concordance greater than 0.8. This experience should provoke reflective thinking for DA developers, digital health scientists, regulators, health technology assessors, and payers.

## 1. Introduction

### 1.1. Context Analysis

Access to essential healthcare services represents a core challenge, politically and professionally, for both developing and developed countries [1,2,3]. According to the World Health Organization in December 2017, 50% of the world’s population lacks access to essential healthcare services [2], Therefore, there is *limited access* to appropriate basic health counseling for the most frequent, common symptoms, which is an ideal time for making the decision regarding the need and type of health care. In developed countries, there is an *inappropriate use* of healthcare resources, which may lead to further difficulties accessing services for those most in need [4,5,6]. In the USA, several reasons for inappropriate use have been identified, including the lack of a proper primary healthcare system that responds to basic health concerns and access difficulties to the appropriate level of care due to multiple structural, financial, and logistical barriers [6]. Additionally, healthcare executives mention that the inappropriate use of the emergency department (ED) for low-severity issues is one of the most critical problems EDs face nowadays [7]. Inappropriate use of health resources is an opportunity-cost loss, as attending low-severity cases at EDs precludes using human knowledge and expertise in other more severe and complex cases. Moreover, the COVID-19 pandemic has shown an increased *demand in ED visits* [7], adding to the already difficult circumstances given the trend towards a decreasing workforce due to shortage or burnout [7]. A shortage of doctors and nurses is now a global challenge, with an expected deficit of 18 million by 2030 [8]. Further access challenges to basic healthcare services are seen in lengthy waiting times and patient–physician miscommunication [9]. *Waiting times at ED* are one of the most frequent complaints registered by users [7,9]. Scientific societies for emergency medicine have set up appropriate waiting times according to the level of severity of the problem (ranging from immediate attention to several levels of “reasonable” waiting times according to risks and acuity) [10,11,12]. However, these times are not always kept [10]. Another issue is *patient–physician miscommunication* which may lead to suboptimal care (including harm and death), decreased understanding of the diagnosis and disease management, and difficulties in shared decision-making [13,14]. Some causes of miscommunication include age-related challenges, reduced capacity or disability, poor language, communication skills, cultural barriers, and literacy and health literacy [13,14]. Therefore, clarity in the written and spoken language at medical encounters is warranted and requested [1].

Besides the current weaknesses related to appropriate care access, traditional health systems are challenged by a shift in attitudes and behaviors of citizens towards greater *consumer engagement and empowerment* in healthcare decisions. A survey on consumer priorities in health care showed that consumers want access to care when, where, and how bests suit them; to comply with these expectations, they are using technology [15], e.g., self-diagnosis with search engines. A survey administered to 1013 United Kingdom (UK) adults revealed that Google was the first point of reference for people as a primary go-to tool when feeling unwell or experiencing potential illness symptoms. Importantly, the core reason for doing so appears to be in response to the difficulty in making a GP appointment [16,17].

All the aforementioned challenges for current traditional health systems, along with the changing trends of society, are pushing health systems to rethink the way health care is organized and provided. New care models are required to ensure quality and access to health care for patients. Furthermore, and to achieve this, costs, behavior changes, and technological progress must be effectively managed. A transformational change in the way health care is organized and provided through a shift towards a patient-centered healthcare paradigm is one of the best options [17], and as seen during the pandemic, emerging digital health technologies have the potential to drive forward said change [18,19]. Digital health technologies are perceived as the tool helping to face demographic challenges (and their associated increasing care demand), to maintain health systems as economically sustainable, and to satisfy citizen expectations [17,20,21]. Moreover, they are being proposed (and starting to be used) to improve access to healthcare services [22] and, for some, decrease miscommunication problems [14]. 

Web-based tools and digital applications (DAs) for counseling when a symptom appears have been developed as first aid for patients and citizens [22]. The idea behind these tools is both to empower citizens in the self-management of their health and to properly drive potential users to healthcare resources when needed (and discourage the access of health care if there is no need). These tools are used as a triage tool (advising the citizen of a course of action: self-care, making a general practitioner appointment, or going to an emergency department) or as a symptom checker (which gives a presumptive diagnosis to the user based on their reported symptom/s) [22,23,24,25].

Though efforts have been made to introduce digital technologies (including DAs) in healthcare systems, resistance in uptaking innovation for breakthrough medical technologies has been seen in the past [26]. Likewise, digital technologies have been developed and updated constantly, which leads to a development uptake gap as healthcare systems are unable to implement these new innovations as soon as they are released. Moreover, most of these innovative DAs are developed by start-ups that face the challenges of introducing technologies aimed at changing the healthcare “status quo” under Innovation + Development, regulatory, assessment, and healthcare adoption requirements designed for very different types of technologies (i.e., medicines, medical devices, IV diagnostics) and for an “outdated” healthcare paradigm (i.e., mostly in person, mostly non-patient empowerment), which hinders their adoption.

For any innovation, the path to develop and move from a conceptual idea to a real product to the market follows specific steps well known in the process of management of innovation, including development of the product, intellectual protection, proof-of-concept and clinical validation, regulatory approval (CE mark in Europe), healthcare system coverage/reimbursement, and healthcare adoption based on the value the new technology brings. For each step, different agents act in deciding the go/no go for the technology. The recently issued EU Medical Device Regulation (MDR) [27] considers digital health technologies, including DAs, as a medical device (MD). Under this regulation, the Notified Bodies (NB) are the institutions that, upon reviewing the results from clinical studies and other technical information, grant access to the European market (CE mark). The CE mark is necessary but not always enough to obtain health authorities’ reimbursement in Europe. Health Technology Assessment (HTA) institutions assess the value of innovative technologies for a specific healthcare system using a predetermined set of criteria and methods, producing recommendations for decisions on coverage/reimbursement by healthcare authorities [28,29]. Finally, adoption of an innovation by the healthcare provider will depend on a myriad of factors (e.g., how the innovation is going to be financed, acceptance by users). Trying to prove value and reach the market for some DAs is filled with obstacles and challenges, especially for start-ups and small and medium enterprises (SME). This process becomes more challenging when regulatory and healthcare systems are still learning how to manage specific disruptive innovations.

The objective of this paper is to present the development and interim comparative effectiveness evaluation of a novel DA called WtsWrng (WW), according to the current requirements for new technologies to access the European market. In doing so, we also aimed to show the challenges faced and the novel methodological approaches used to evaluate WW.

### 1.2. WtsWrng (WW) DA: Why and for What Purpose

In the initial phases of developing WW, the first step was the identification of the unmet need, the target population, and where the technology will be used in the pathway of care. From the beginning, it was clear that WW aimed to be a tool to offer citizens quicker and more agile access to answers to their basic health concerns and to be placed as an additional virtual access option for current in-person health care. It was aimed to empower citizens in their decisions about how to proceed when a symptom appears. Though few similar online and smartphones DAs already exist [22], weak scientific evidence of their effectiveness is available [30]. Additionally, all available DAs are built using written and oral language systems, which are inherently subject to miscommunication in the patient–physician encounter, often leading to medical errors as mentioned above [1,13,14]. Moreover, using written and spoken language also limits the worldwide scalability of the product, even when using artificial intelligence (AI)-based translators. WW creators developed a solution to overcome these challenges by both designing a DA using the universal language of images to interact with citizens and aiming to generate strong evidence of its effectiveness comparing WW with the current standard of care at the ED and primary care center (PCC).

**Figure 1:**
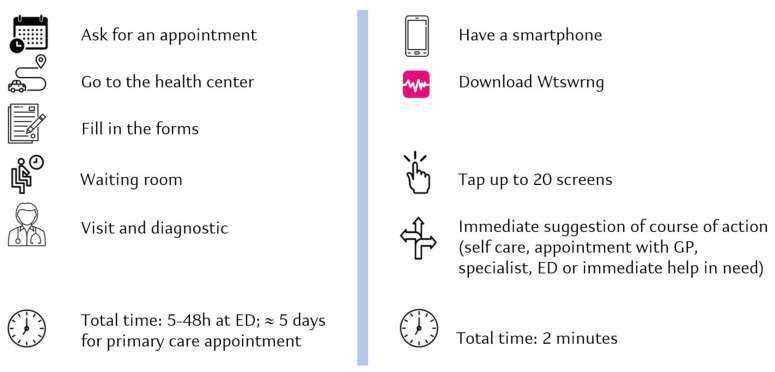
Patient journey: conventional care versus WtsWrng. ED: Emergency Department; GP: General Practitioner.

The WW DA is a digital triage, symptom checking, and monitoring solution that can be downloaded on any smartphone. Given a reported symptom, WW provides suggestion on basic, non-prescriptive behavior using only images, intentionally avoiding the use of written or voice language. This unique feature makes WW a disruptive DA since, as of today, we are not aware of any triage DA that interacts with users via images only. The user navigates through a series of encoded drawings regarding his/her symptoms and, after a reduced digital anamnesis (maximum 2 min), suggests a course of action (self-care, appointment with general practitioner, specialist, emergency department, or immediate help is needed). Figure 1 shows the patient journey of WW compared with the current access to care.

**Figure 2:**
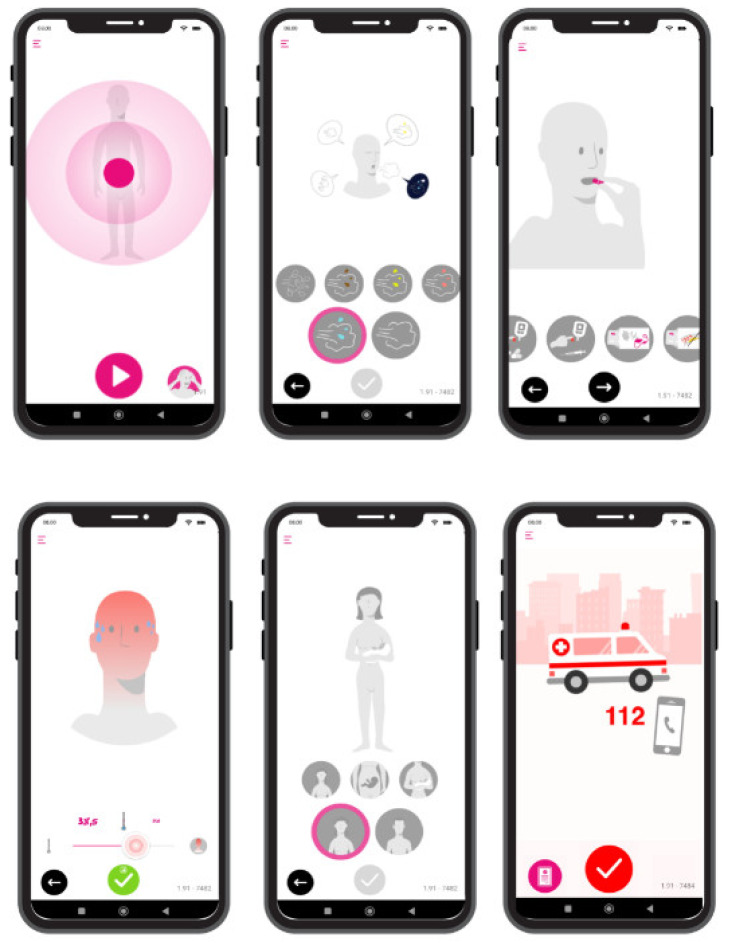
Sample of images shown on WW screens to interact with citizens.

WW displays the 20 most frequent symptoms that lead to an ED or a non-scheduled PCC visit on the user’s smartphone [16,31,32]. The user selects the symptom and scrolls through screens, where different images appear, reflecting aspects of usual anamnesis performed by clinicians in person to help patients in thinking about a potential presumptive diagnosis linked to their initial symptom/s. With the time reduced, digital anamnesis leads to a set of presumptive differential diagnoses. These presumptive differential diagnostics are kept in the background and are not visible to the user. They are used to stratify users by risk of severity and, consequently, suggest the most appropriate course of action. Figure 2 shows the WW interface.

## 2. Methods: WtsWrng Development and Evaluation

### 2.1. Building the First Prototype: Ensuring the Accuracy of WtsWrng Decision Trees and the Subsequent Algorithms and Drawing Design 

A total of 20 symptoms were identified as the most frequent reasons for attending the ED and non-scheduled PCC visits. These symptoms come from a mix of different sources: the results of a field study performed at a hospital (Clinic Barcelona University Hospital—CB) and PCC in our country (Catalonia/Spain), from selected literature reviews [16,31,32,33] (to ensure transferability), and from an expert panel consisting of senior emergency physicians that discussed the symptoms to be included. The symptoms digital anamnesis led to 336 presumptive differential diagnoses, including diagnosis for some treatable rare diseases.

A multidisciplinary team reviewed the medical literature, looking for evidence-based symptoms guidance that is used in primary care and emergency room triages [34,35,36,37,38,39,40], and these evidence-based resources were used to develop the core decision trees (i.e., from each of the 20 symptoms triggering a visit to doctor, to the 336 presumptive differential diagnoses) [34,35,36,37,38,39,40]. Decision trees were then tested using clinical simulation in more than 6000 “near-live” clinical cases, as suggested by some authors [20]. The result of this technical validation was the basis to create the training rules to design a predictive machine learning-authored algorithm, with the aim of reducing the number of questions required until reaching the final WW suggestion on the course of action, as the number of users grow, to a similar number to that used during a standard/conventional presential or phone clinical anamnesis. 

As previously mentioned, WW only uses images. To create the images, a review of international comics (from different continents) to select images of signs (e.g., pain) that could be clearly understood by different cultures was undertaken. The understanding of a first version of images was tested by a small convenience sample of people (colleagues, friends, family) from different cultures (Caucasian, Hispanic, black people). To date, WW includes more than 5000 encoded original and protected drawings.

### 2.2. Usability and User Experience

A small-scale usability (i.e., observing how users interact with the product) and user experience (UX) test was conducted to identify usability issues, and to assess use and acceptance. The test was performed on a sample of 100 citizens attending the ED of a hospital (n = 50) and its linked PCC (n = 50). The test was performed in-person by a field researcher. A questionnaire was designed and used to capture demographic characteristics (country, native language, gender, age), digital literacy (literacy, use and type of digital tool-smartphone, computer, tablet, smart watch, wearable, none; level of DA use, online buying, social network use), and UX (WW usefulness, intuitiveness, design liking, understanding of images, visibility of images, easiness of images selection, future use of WW, and recommendation of WW to others).

### 2.3. Clinical Validation of WtsWrng: From Fast-Track Early Scientific Advice from NICE on WW Evidence Generation Plan to the Study Design, Agile Iterative Development, and Agile Iterative Interim Analysis

A cross-sectional, single-arm clinical trial was designed to test the accuracy and precision of WW [41,42]. The study design aimed to demonstrate that WW (i.e., the intervention) is non-inferior (i.e., at least equal or better) in precision and accuracy than the tools currently used by a hospital ED to triage (i.e., Structural Emergency Triage. Andorra Triage Model-MAT) and to diagnose patients (i.e., the diagnosis written on the ED clinical record at patient discharge). These two comparators are considered the “gold standard” in this study. The study protocol was submitted to NICE (National Institute for Health and Clinical Excellence, UK) to obtain early scientific advice, under the fast-track review guidance for COVID-19 studies (request SA593) [43], leading to the inclusion of some of the advice received in the protocol. The final clinical protocol obtained the approval of the Ethics Committee of the Clinic Barcelona University Hospital (CB), where the evaluation was performed (Reg. CB/2020/0087).

WW was tested on a sample of consecutive patients attending the ED of CB from October 2020 to July 2021, having met the predefined inclusion criteria (i.e., patients over 18 years old, triaged to ED levels III, IV, or V, and conscious in order to use WW with a smartphone). Patients triaged to levels I and II were excluded from the study since they were unconscious or very severely ill or injured in need of immediate attention and, therefore, are unable to properly use WW.

A field researcher attended the ED of the hospital and asked for voluntary participation. The patients were given a smartphone with WW and were asked to use it. Final suggestions for the user course of action were masked at this point (i.e., no suggestion on course of action was displayed on the screen as the end result). Written informed consent was obtained from all participants.

The triage system of the CB’s ED uses a nurse-driven computer system (MAT [44]) that sends patients to different levels of care depending on five degrees of severity, as defined internationally [45]. To assess triage accuracy and the precision of WW with the ED system at the hospital, based on clinicians’ suggestions, we considered that level III corresponds to the suggested course of action to go to hospital/specialized care, level IV corresponds to going to primary care (i.e., non-emergency cases that do not need to go to a hospital ED but to a PCC), and level V corresponds to self-managing the symptoms. Given a symptom, triage accuracy was calculated by means of comparing the percentage of patients correctly triaged by WW, given the results from the ED triage system. Additionally, to test triage precision, the percentage of patients correctly triaged by WW was compared to the results from the level assigned (i.e., III–V) by the ED triage system at the CB, by calculating the observed concordance, Kappa, and B statistics. 

**Figure 3:**
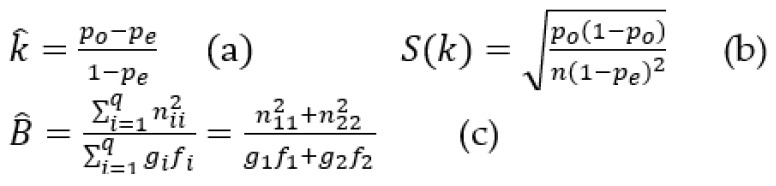
Kappa and B statistic calculation formulas. Legend: where n is the total number of observations, $n_{ii}$ is the $n_{11}$ and $n_{22}$ representing the number of agreements between hospital and WtsWrng; $g_{i}$ is the $g_{1}$ and $g_{2}$ representing the total diagnoses identified and not identified by the hospital; $f_{i}$ is the $f_{1}$ and $f_{2}$ representing the total diagnoses identified and not identified by WtsWrng; $p_{o}$ is the proportion of the observed agreements between the hospital and WtsWrng $\left(n_{11}+n_{22}\right)/n$; and $p_{e}$ is the expected agreements $\left(g_{1}g_{2}+g_{2}f_{2}\right)/n^{2}$ assuming independence between the assessment of hospital and WtsWrng.

Precision and accuracy between the presumptive diagnosis given by WW and the clinical record diagnosis at patient discharge were also analyzed. As first choice, the ICD-10 coded diagnostic at discharge from the ED was extracted from the administrative database of the hospital designed for billing purposes. Their ICD-10 codes were considered the “gold standard to compare”, with presumptive diagnosis given by WW (also coded with ICD-10). During the interim analysis, inaccuracies in the administrative database were detected; therefore, the clinical records at discharge were also reviewed to have the final diagnosis given to the patient for comparison. The diagnostic precision of WW was then studied by means of the concordance between the diagnostic of a patient at ED discharge (from clinical records) and the first 5 presumptive diagnoses provided by WW. The observed concordance, Kappa, and B statistic were applied to the 12 most frequent diagnostics (defined as n ≥ 9 patients) at hospital discharge, to observe potential differences between the results when applying these statistical parameters. Kappa is the most used parametric statistic to assess concordance between a diagnostic health technology and the gold standard [46]. The estimated Kappa is a variable that follows a normal distribution with mean *k* in formula Figure 3a and standard deviation *S*(*k*) in formula Figure 3b. To properly use Kappa as a value statistic, it requires both a high and similar number of observations in $n_{11}$ and $n_{22}$, as well as similar numbers in $n_{21}$ and $n_{12}$ (see Table 1). The B statistic in formula Figure 3c is a non-parametric statistic that can also be used to assess concordance between a diagnostic health technology and the gold standard; it has as advantage over Kappa in that similar numbers are not required in $n_{11}$, $n_{21}$, $n_{12}$, and $n_{22}$ [47], as observed in our study. Figure 3 shows the Kappa and Beta statistic calculations, while Table 1 shows the theoretical distribution of subjects by diagnostic sources (WW versus ED clinical record at discharge) and response category.

Finally, presumptive diagnosis sensitivity and specificity of WW compared with the diagnostic at hospital discharge were calculated for the 12 most frequent diagnostics attending the ED.

At the time of clinical testing, WW was in a development stage (Technology Readiness Level 6) [48]. This meant that an agile iterative analysis was necessary, where quick rounds of testing–redesigning–testing were conducted [49]. It is also important to mention that the study was performed during the COVID-19 pandemic; therefore, evolving symptoms from this disease were also introduced into the decision trees that fed the algorithms during the study period.

Stata 13 software [50] was used for calculation purposes. Nevertheless, the B statistic was hand-calculated using specific formulas since Stata does not include a program for this purpose.

### 2.4. Protection of WW

The European Union granted WW access to IPA4SME, a program devoted to innovative European SMEs, to analyze ways to protect any asset [51]. Following the results from the audit, different protection modalities were performed.

## 3. Results

### 3.1. Usability and User Experience

Of the 100 users, 60% of the sample were women. Age ranged from 18–29 (15%) to more than 71 years old (21%, including a 94-year-old). Participants born in Spain comprised 72% of the sample, with the rest (28%) non-Spaniards (Lebanon, Pakistan, Sweden, and Latin-American countries). Native language included Spanish (n = 52), Catalan (n = 38), and a variety of other languages (Galician, Italian, Urdu, Portuguese, Swedish, English). One person was not literate. Smartphone users comprised 99% of the sample, while one person did not use any type of digital device. Of the 100 people using smartphones, 80 used DAs in their smartphone, 79 of 100 people they buy things online, and 60 of the sample used social networks. Based on a Likert Scale from 1–10 (1 not useful at all, 10 very useful), 81 people (79%) scored WW over 7, which shows that WW is perceived as a useful tool. They also reported a good understanding (60%), visibility (85%), and easiness in selecting the images on the smartphone (87%). Moreover, 90 participants (88%) liked the design (images) and 83 (81%) mentioned their willingness to use WW when available. Lastly, 87 people (85%) mentioned that they would recommend WW to others.

### 3.2. Clinical Validation: Agile Iterative Interim Analysis

A total of 567 patients who met the inclusion criteria were enrolled in the study. There were 117 patients excluded due to failures in the transmission of data to the website (transmission failures happened at different days and times during the study period). Of the remaining 450 patients, 45 did not have a diagnosis at discharge in their clinical record (i.e., they usually had the same symptoms that originated the visit to the hospital ED) and were excluded from the analysis. Finally, from the 20 more frequent symptoms that trigger doctor visits, those symptoms with fewer than 10 patients were excluded from the analysis, accounting for a further 23 patients; the exclusion of these patients was made to avoid infra or overestimation of WW effectiveness. The data of the remaining 382 patients were finally included for the assessment of WW’s precision and accuracy of triage and presumptive diagnosis. Table 2 shows the demographic characteristics of the 382 participants in the clinical study.

**Figure 4:**
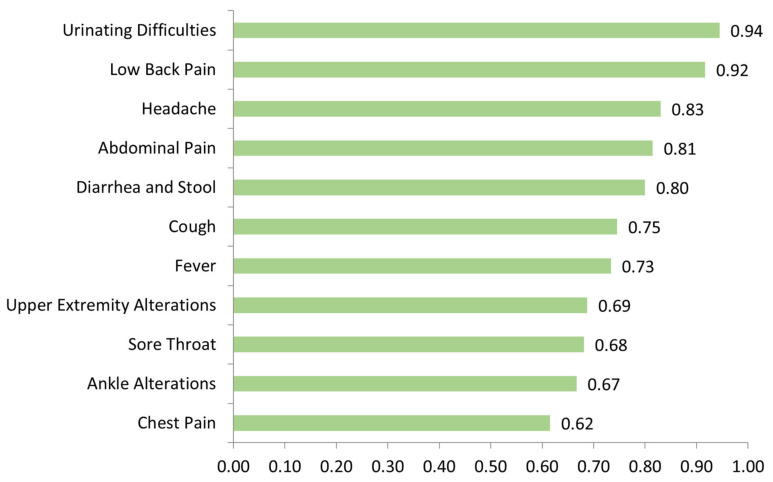
WW triage accuracy (from 0 to 1, with 1 equal to 100% accuracy) for the most frequent symptoms attending ED.

A WW triage accuracy ranging from 0.62 to 0.94 for the most frequent symptoms attending ED was observed. Figure 4 shows the results of triage accuracy by symptom. Triage precision showed an observed concordance ($p_{o}$) of 0.78, a Kappa of 0.39, and B statistic of 0.81. The B statistic was shown to be closer to the observed concordance than Kappa.

WW precision in presumptive diagnosis for the 12 most frequent diagnoses across the studied patients by means of observed concordance, Kappa, and B statistics is shown in Table 3.

The observed concordance ($p_{o}$) for the 12 most frequent diagnostics attending hospital ED fluctuated between 0.4 to 0.97; for 8 of the 12 diagnostics, the observed concordance was over 0.8. When using Kappa, the results ranged from 0.005 to 0.37, while the B statistic ranged from 0.36 to 0.97, which is closer to the observed concordance ($p_{o}$). The B statistic continued to be closer to the observed coefficient of concordance than Kappa statistics. The sensitivity and specificity of WW were found to exceed 0.8 for 3 and 8 of the 12 most frequent diagnostics, respectively. Table 4 shows a summary of the results.

### 3.3. Protection of WW

Following the recommendations given by the IPA4SME program regarding intellectual protection modalities, WW was registered as a European Union Trademark/OHIM (#016480006). Main images were protected, while trade secret and copyright under Directive 2009/24/EC were also used as other barriers to disclosure. WW was also registered at the Beneloux Office for Intellectual Property (iDEPOT # 113467).

## 4. Discussion

WW is the result of a compilation of digitalized evidence-based clinical decision trees that use supervised (i.e., as input data are fed into the algorithms, healthcare professionals check the output, with the weights of the algorithms adjusted until the model has been fitted appropriately; this is a cross-validation process activity) machine learning algorithms [52] to provide a disruptive (i.e., image-driven) clinical anamnesis and symptom triage to aid citizens in their decision regarding the need to see a healthcare professional, when and where, and to monitor any changes in the course of the suspected pathology, redirecting them to the appropriate level of care. WW aims to disrupt [53] and to be used as the first resource globally for people that have a health concern (symptom), by using their smartphone. WW’s quick and easy access can contribute to overcoming some of the current challenges that the traditional health system faces in line with some suggestions of systemic transformation for health systems [26]. Moreover, the new approaches proposed to develop and evaluate WW aims to inspire health stakeholders towards exploring new regulation requirements and policies for this type of technology.

Access to basic healthcare counseling, due to a lack of infrastructure [2,6] or long waiting times when an acute health problem appears [7], is a structural problem of current health systems. WW is easily accessible for smartphone users (83.40% of the world’s population owns a smartphone [54]) and provides a suggestion of action in a short space of time (2 min), which can contribute to overcoming accessibility problems [54]. Importantly, although WW is based on authored algorithms, an API (Application Programing Interface) web can be developed to allow integration and compatibility with health system requirements, minimizing the technology country’s adoption barriers. The results of our usability and UX initial study show a good acceptance of WW with a high willingness to use WW when available (81% of surveyed people) and disposition to recommend it to others (85% of surveyed people)

Inappropriate use of healthcare resources is also a challenge faced by health systems. In the UK, 61% of the visits at a hospital ED and PCC were not necessary [4], which represents an avoidable expenditure of EUR 900 million in 2020 [4]. In the USA, between 13.7% and 27.1% of visits to a hospital ED and PCC are not necessary, accounting for unnecessary expenditure of USD 4.4 billion yearly [5]. The results from the WW’s interim analysis in a real-life clinical setting showed a good global precision as a triage tool (B statistic of 0.8, global observed concordance of 0.78). These results can position WW as a tool to guide a more appropriate citizen decision for using healthcare resources and, therefore, with the potential to avoid unnecessary expenditure. However, this claim may be disputed as some authors mention that the current triage DAs do not comply with their claim to reduce demand [55]. Conversely, others mention that it is not clear if the increase in demand of services observed may be due to the emergence of an unmet need or a supply-induced demand [56]. A specific study to test the impact of WW in decreasing demand and improving efficiency in healthcare resource use in a large real-life study, as suggested by some authors [57], is already planned.

A lack of a proper primary healthcare system that answers basic health concerns has also been identified in the USA as a reason for inappropriate ED use [6]. In this country, a review of 115,081 medical records showed that the avoidable top five ED disorders were mild complaints that a PCC can assess as a first contact [17]. In our study, WW has demonstrated good precision in identifying those patients that would have been more appropriately directed to primary care (0.95 observed concordance and 0.94 B statistic when comparing a hospital triage system—those patients directed to level IV—with WW results suggesting going to a primary care physician). Finally, research suggests that current DAs that are effective in triaging are not the same as those that have been shown to be good at diagnosing [55]. In this study, WW showed good specificity (over 0.8) for 8 of the 12 most frequent diagnoses attending ED, which compared favorably with similar DAs [58]. These results show the good performance of WW as both a triage and a presumptive diagnosis tool.

WW operates using images in its interaction with the user, aiming to both overcome the miscommunication problems linked to the current spoken and written medical encounters with patients and answering a rising social trend. Regarding the former, creative solutions are demanded [1]. As for the latter, images are identified as the tools to be used in future communication, including medicine. A current global trend is the rising use of digital tools using images (e.g., video games, where the number of users is expected to reach 2829.8 million by 2027) [59,60]. In the medical field, some professionals and authors are already claiming that they can facilitate communication in the medical field because of the avoidance of the ambiguity of words [61,62]. The usability and UX test performed for WW in a sample of multicultural patients, with a different level of literacy, in a real-life clinical setting showed good visibility and understanding of images, which were also easy to select on the screen. Nevertheless, following users’ suggestions, some images were improved at this point (more than 44 improvements were made).

Besides the societal trend to use more and more images in communication, there is also a trend towards both citizen empowerment in healthcare decisions [63] and immediateness in receiving an answer to basic health concerns, skipping an in-person visit that may not be necessary and takes time. As a digital health tool, WW overcomes the in-person, non-patient-centered approach to healthcare provision prevalent in traditional health systems [63]. Immediateness is answered nowadays by using digital tools. Currently, 6 out of 10 Europeans citizens go online when looking for health information; health care is the third-largest web activity across all generations [17]. According to the 2014 edition of the McKinsey report (N = 3000 consumers surveyed), for more than 90 respondents, DAs and websites are more effective ways to perform quick consultation activities than in-person or even phone consultations [63]. Another study showed that 59% of US adults have looked online for health information to make a decision or course of action; after digitally consulting, 38% thought that it was something they could take care of at home and 46% thought that they needed attention from a medical professional, while in 11% of cases, it was both or in-between [16].

Any related health DA devoted to suggesting, informing, or driving a course of action will have health consequences if the users act according to the information provided. Therefore, the credibility of the information used by any DA is critical [64]. This means that the background where the tool runs, as well as its clinical assessments, must be effective and of the highest-possible quality. In the case of WW, evidence (evidence-based guidance), experience (participation of healthcare professionals), and technology (supervised machine learning) were all considered in its development, ensuring minimal deviation from proper triage and diagnosis.

One of the evaluation activities needed to advance any emerging DA, before its market release and to promote user uptake, is to prove its effectiveness in a clinical setting, and its comparative clinical effectiveness with the current standard of care. How to assess DAs nowadays is a matter of research. There are more than 40 assessment frameworks for DAs in an advanced stage of development [65], 21 for eHealth [66], and some author suggestions for types of studies recommended depending on the maturity of the technology [48]. Nevertheless, we did not find any internationally accepted high-quality standard guidance to be used in the design of the WW study which is in an intermediate stage of development (TRL6, TRL ranges from 1–9, a technology with TRL = 9 means that it is in the market). Therefore, we used a study design and an analysis method that better suits the characteristics of WW (i.e., DA, in developmental stage, need for a proof of comparative effectiveness), while approaching the methodological standards required when comparing two technologies for the same purpose. The cross-sectional single-arm clinical trial conducted followed the agile software movement, where incremental, iterative measures are taken, allowing for both empirical feedback and tool improvement [49,67]. This method is one of several that overcomes the challenges that standard methodologies use to test non-digital technologies (e.g., randomized controlled trials with two cohort groups) when they are used to evaluate technologies characterized by a fast evolution and update [20]. This method allowed us to cope with the unprecedented circumstances of COVID-19 during the trial. WW was able to immediately adjust the decision trees and algorithms at the same pace that new COVID symptoms were described. This was important since symptoms associated with COVID-19 were non-specific (i.e., they can be associated with different pathologies), so a proper identification and inclusion in the decision trees, and final classification by level of severity to suggest the right course of action to the user, was needed.

Choosing the right comparator is also a key quality requirement when performing a comparative assessment of technologies [29]. In our study, the diagnosis written in the ED clinical record at discharge was used as the “gold standard” comparator. When reviewing the clinical records, some non-appropriate reporting was found (i.e., in 45 clinical records, only the symptoms were reported without any diagnosis). Since the end of the 20th century, health information systems have evolved substantially; however, in some settings, a proper coding of diagnosis by doctors still lacks accuracy. If the “gold standard” to compare with the new tool (i.e., WW) has weaknesses, the comparative results may be erroneous, thus penalizing the new tool. This is especially relevant nowadays since real world data (RWD) are more frequently claimed to be used for evaluating technologies. Health systems produce abundant RWD, but its use in research is challenged by both the quality of these data as well as their lack of parameterization. Common data models and standards are still needed to use RWD in a robust and transparent way [68].

To ensure the highest possible quality in the methods used, we explored which statistic was better in assessing WW performance. Traditionally, assessment of concordance of two measurement systems is performed using the Kappa statistic. However, this statistic should be used when the data comply with several assumptions (i.e., normal distribution, a high and similar number of observations between observed agreements and disagreements, marginal values are uniformly distributed) [69]. The assumption that the marginal values are uniformly distributed is not the case in our study, leading to a high weighting of the discordant values when calculating the concordance [47]. This situation underestimates the Kappa results for WW. Several authors investigated how to assess concordance under Kappa limitations [69,70,71,72]. These studies compared different estimators of concordance including Cohen’s Kappa, B Statistic, prevalence index (PI), Gweet’s AC1 index (AC1), Matthew, prevalence-adjusted and bias-adjusted Kappa (PABAK), and Delta; it was found that the B statistic gives the closer interpretation to the real concordance (i.e., observed concordance) [69]. The results from our analysis showed that B statistics gets closer to the observed concordance than Kappa. Therefore, the B statistic is a superior statistic to show the real precision of WW as a triage and diagnosis tool.

Several limitations may exist in the assessment of WW comparative effectiveness. We did not use a validated instrument to test WW usability and UX tests. Therefore, the results obtained could differ from another sample of respondents should a validated questionnaire be used. Nevertheless, during the clinical evaluation, minor suggestions were given by users about the WW design and performance. Another limitation during the usability test was the digital literacy measurement. We did not employ a validated questionnaire, opting to use a direct question with several potential answers, which included the range of digital tools most frequently used by society. However, although we would have used a validated questionnaire, all measurements of digitality are subject to change because of technology trends. Moreover, considering the Agency for Health Research and Quality (AHRQ) check list mentioned in Semigran (2015) [73], WW fulfills almost all the requirements to be a health technology suitable for populations with all grades of literacy. Finally, by conducting this first evaluation of WW among patients attending a hospital ED, the results regarding precision and accuracy may only be applicable to those users who have moderate–severe symptoms and not for those with moderate–mild symptoms who must be directed to primary care. Looking at the precision by level of triage, there is a good concordance for the level attending who have moderate–mild symptoms (level IV = B statistic of 0.94), which may indicate that WW triage precision for this type of population is good. However, these interim results should be confirmed in a future study including patients attending emergency primary care.

The promise that WW presents in overcoming both current health system challenges and the inherent limitations of traditional evaluation methods applied to DA technologies such as WW have been previously described and discussed. However, other challenges exist in the race of DAs to reach the market, for example, in legal terms. For any innovative technology, some level of protection to guarantee a high probability of appropriate market access is advisable. Ensuring proper protection for a DA is a key to accessing potential future investments to further develop, operate, and ensure a proper use at DA launching. One of the most reliable forms of intellectual protection is via a patent. However, in the EU, patentability is not available for most DAs. The lack of a strong protection strategy for these types of technologies renders them at risk to be copied by financially stronger developers or companies. This is especially relevant for start-up companies, one of the main producers of these type of technologies and who typically have tight budgets. Regulation represents another central challenge. The new EU medical device regulation (MDR) considers DAs to be a medical device (MD) [27]. However, WW is placed in a gray area when considering the definition of MDs by the new MDR. WW does not provide the user with a diagnosis, which is solely stored in the system. Therefore, although it is a tool linked to health care, it may not be considered strictly as a MD by some NB. In fact, one NB in the EU considered WW not to be a MD, instead considering it to be in a gray zone. It could be the case that other NBs consider WW as a MD. Not having a regulatory granting may be detrimental for the product since potential users are asking for some form of quality certification before using this type of product [74].

Besides the CE mark, compliance with other regulatory requirements (i.e., ISO13485) is needed in the EU for software. Reaching the CE mark and obtaining and maintaining an ISO is extremely difficult, requiring intensive human and economic resources. A question to ponder is whether the “one-size-fits-all model” that is currently asked for any type of MD, under the new MDR, should still be kept for emerging DAs, or if a new model specifically designed for a DA that stratifies regulatory requirements by type of digital technology (including DAs) and technology readiness level (TRL) of the product should be created.

Finally, another challenge for innovative DAs are the EU evidentiary requirements by public payers. In Europe, having a CE mark for a MD is necessary for market entrance; however, it does not guarantee adoption by healthcare systems. Proving that the MD performs at least equally or better than the standard of care is demanded by payers. Currently, there is no clear, standardized guidance on how to clinically assess the wide range of DAs. As already mentioned, a total of 45 frameworks to assess mobile applications for medicine have been identified by a systematic review [65]. Moreover, different challenges have been described in applying the traditional clinical research methods to DAs [20]. In the search to look for a more appropriate process to assess DAs, Germany has recently implemented the DiGA process for DAs with a CE mark class I and IIa. DiGA was born as a fast-track to market for those DAs with demonstrated benefits or with great potential. When a DA enters DiGA, the reimbursement is guaranteed to prove that the benefits claimed are obtained after one year of implementation. At the end of this period, a new reimbursement level is negotiated or withdrawn in the event that the DA fails to prove beneficial. DiGA does not have explicit public methodological guidance to assess the DA; methods and outcomes to be assessed are set up case by case through discussions between the DA producer and BfARM, the regulatory agency for MD in Germany [75]. While DiGA is a good first step to help advance a DA to market, the short experience shows that several weaknesses in the model exist [76]. Other initiatives also exist in Europe to guide the assessment of DA to obtain reimbursement. One is the so called “Belgium pyramid” that increases the complexity of scientific requirements that prove DA benefits linked to the level of its classification (class I, II, III) [77]. Similarly, the National Institute for Health and Clinical Excellence (NICE) has recently issued a new version of its Evidence Standard Framework (ESF) for digital health technologies, which classifies technologies by their potential risk to service users and systems according to their intended purposes. Depending on the classified tier, the DA will have to comply with different evidentiary requirements [78]. Here, a challenge may appear for WW due to a contradictory position between a notified body (which considers WW not to be a MD) and NICE which, through its ESF, would consider WW as a MD and classify it in tier 3, asking for the most comprehensive and strict fulfillment of evidentiary requirements. As such, the evidence generation approaches that are appropriate for DAs, to fulfill most health authorities’ requirements, is a theme of current debate in European member states. The methods used in our study for assessing the comparative effectiveness of WW, following suggestions from other authors [49,53], may be used as an inspirational insight for researchers and health technology assessment scientists in developing more appropriate methods for the evaluation of digital technologies at different TRLs.

## 5. Conclusions

Digital health is among the key phenomena driving the next cycle of transformation of health systems. Digital technologies—DA, wearables, and software algorithms—have the potential to offer easier and more agile access to support a technology-enabled health system in which care interactions with citizens, for some medical encounters, are moved away from traditional settings by encouraging citizens to manage their own health and mild health problems. In turn, clinicians will optimize their time using artificial intelligence (AI) solutions as a support to their daily work. Accordingly, digital health will enable “using humans for the hard stuff and leaving the basics to machines” [79].

Nevertheless, the proper development and clinical testing of the benefits of emerging DAs is a challenge in traditional healthcare systems. Weaknesses in the available RWD of comparators, lack of strong protection rules in some jurisdictions, requirements to use traditional study designs for technologies that are continuously evolving, and/or lack of accepted international standards for granting the access and coverage for DAs into health systems are obstacles to developing innovative DAs, especially for start-ups who are the main developers of these types of technologies.

WW is a promising and disruptive DA, with a high potential scalability and social impact that will allow citizens worldwide to access advice and answers on what to do when faced with a basic health concern. To prove its value from inception, it has been required to overcome challenges which characterize most current health systems (i.e., information system design and content, regulatory and assessment evidentiary requirements) with novel approaches for evidence generation that allows for its agile, and patient-centered, development. Though still not marketed, and in need of advancement in terms of WW maturity level, the experience developing WW should be a matter of critical, reflective thinking for DA developers, digital health scientists, regulators, health technology assessors, and payers, in the quest for designing an appropriate pathway to a market tailored to DAs.

## Figures and Tables

**Table 1:** Theoretical distribution of subjects by diagnostic sources and response category.

		WtsWrng		
		NO	YES	Total
**Hospital**	NO	$n_{11}$	$n_{12}$	$g_{1}$
	YES	$n_{21}$	$n_{22}$	$g_{2}$
	Total	$f_{1}$	$f_{2}$	

**Table 2:** Characteristics of sample (N = 382).

	n (%)
Gender	
Female	179 (47)
Male	203 (53)
Age	
18–29	43 (11)
30–39	37 (10)
40–49	35 (9)
50–59	67 (18)
60–69	78 (20)
70–79	80 (21)
80–99	42 (11)
Nationality	
Spain	312 (82)
Other European countries	14 (4)
Latin American countries	47 (12)
Others	9 (2)
Employment status	
Housekeeper	2 (1)
Self-employed	6 (2)
Student	13 (3)
Employed	128 (34)
Unemployed	37 (10)
Sick leave	19 (5)
Retired	177 (46)
Higher education	
Without studies	9 (2)
Primary school	73 (19)
Secondary school	34 (9)
High school	135 (35)
Bachelor’s degree	103 (27)
Master’s degree	18 (5)
PhD	10 (3)
User of technologies (Mobile, Tablet, etc.)	
YES	360 (94)
NO	22 (6)
Use of DA	
YES	248 (65)
NO	134 (35)
Use of health DA	
YES	165 (43)
NO	217 (57)

**Table 3:** Observed concordance, Kappa, and B statistics for the 12 most frequent diagnostics at hospital discharge (ordered for B statistics).

	$n_{11}$	$n_{12}$	$n_{21}$	$n_{22}$	$g_{1}$	$f_{1}$	$g_{2}$	$f_{2}$	$p_{o}$	k	B	Sens.	Spec.
Chronic kidney diseases	371	10	1	0	381	1	372	10	0.97	0.00	0.97	0.000	0.997
Lung cancer	368	5	7	2	373	9	375	7	0.97	0.23	0.97	0.286	0.981
Cellulitis	366	3	10	3	369	13	376	6	0.97	0.30	0.97	0.500	0.973
Gastroenteritis	354	5	16	7	359	23	370	12	0.95	0.37	0.94	0.583	0.957
Heart failure	343	21	16	2	364	18	359	23	0.9	0.05	0.90	0.087	0.955
Urinary infection	332	3	40	7	372	10	335	47	0.89	0.21	0.88	0.700	0.892
Asthma	333	4	42	3	337	45	375	7	0.88	0.09	0.88	0.429	0.888
Low back pain	309	2	66	5	311	71	375	7	0.82	0.10	0.82	0.714	0.824
Renal colic	292	3	80	7	295	87	372	10	0.78	0.10	0.77	0.700	0.785
COVID-19	136	10	162	74	146	236	298	84	0.55	0.20	0.38	0.881	0.456
Other respiratory disorders	144	2	227	9	146	236	371	11	0.4	0.02	0.37	0.818	0.388
COPD	141	1	225	15	142	240	366	16	0.41	0.04	0.36	0.938	0.385

Sens: Sensitivity; Spec: Specificity.

**Table 4:** WW precision and accuracy comparing the first 5 WtsWrng presumptive diagnoses and the diagnosis in ED clinical records at discharge.

	Results (N = 382)
Observed concordance ($p_{o}$)	0.4–0.97 (0.8 in 8 diagnostics) *
Kappa	−0.005–0.37
B statistic	0.36–0.97 (0.8 in 8 diagnostics) *
Sensitivity	0.09–0.94 (0.8 in 3 diagnostics) *
Specificity	0.39–0.98 (0.8 in 8 diagnostics) *

* Numbers in brackets: number of diagnostics, over the 12 most frequent diagnostics at hospital discharge, with a result ≥0.8.

## Data Availability

The data presented in this study are available on request from the corresponding author. The data are not publicly available due to patient data protection.
